# A complex *eIF4E locus* impacts the durability of *va* resistance to *Potato virus Y* in tobacco

**DOI:** 10.1111/mpp.12810

**Published:** 2019-05-21

**Authors:** Vincent Michel, Emilie Julio, Thierry Candresse, Julien Cotucheau, Christophe Decorps, Roxane Volpatti, Benoît Moury, Laurent Glais, Emmanuel Jacquot, François Dorlhac de Borne, Véronique Decroocq, Jean‐Luc Gallois, Sylvie German-Retana

**Affiliations:** ^1^ UMR 1332 Biologie du Fruit et Pathologie INRA, University Bordeaux 71 Av. E. Bourlaux Villenave d’Ornon Cedex CS 20032 33882 France; ^2^ Seita Imperial Tobacco La Tour 24100 Bergerac France; ^3^ Unité de Pathologie Végétale, INRA, Centre Recherche PACA, Domaine Saint Maurice Montfavet Cedex CS 60094 F84143 France; ^4^ UMR IGEPP INRA, Domaine de la Motte BP 35327 Le Rheu Cedex 35653 France; ^5^ INRA‐Cirad‐Supagro Montpellier, UMR BGPI Montpellier Cedex 34398 France; ^6^ INRA‐UR 1052, GAFL Domaine St Maurice – CS 60094 Montfavet Cedex F‐84143

**Keywords:** *eIF4E*, tobacco, potyvirus, *va* resistance, durability, Ethyl methanesulfonate mutant

## Abstract

Many recessive resistances against potyviruses are mediated by eukaryotic translation initiation factor 4E (eIF4E). In tobacco, the *va* resistance gene commonly used to control *Potato virus Y* (PVY) corresponds to a large deletion affecting the *eIF4E‐1* gene on chromosome 21. Here, we compared the resistance durability conferred by various types of mutations affecting *eIF4E‐1* (deletions of various sizes, frameshift or nonsense mutations). The ‘large deletion’ genotypes displayed the broadest and most durable resistance, whereas frameshift and nonsense mutants displayed a less durable resistance, with rapid and frequent apparition of resistance‐breaking variants. In addition, genetic and transcriptomic analyses revealed that resistance durability is strongly impacted by a complex genetic locus on chromosome 14, which contains three other *eIF4E* genes. One of these, *eIF4E‐3*, is rearranged as a hybrid gene between *eIF4E‐2* and *eIF4E‐3* (*eIF4E‐^2‐3^*) in the genotypes showing the most durable resistance, while *eIF4E‐2* is differentially expressed between the tested varieties. RNA‐seq and quantitative reverse transcriptase‐polymerase chain reaction experiments demonstrated that *eIF4E‐2* expression level is positively correlated with resistance durability. These results suggest that besides the nature of the mutation affecting *eIF4E‐1*, three factors linked with a complex locus may potentially impact *va* durability: loss of an integral *eIF4E‐3*, presence of *eIF4E‐^2‐3^* and overexpression of *eIF4E‐2.* This latter gene might act as a decoy in a non‐productive virus–plant interaction, limiting the ability of PVY to evolve towards resistance breaking. Taken together, these results show that *va* resistance durability can in large part be explained by complex redundancy effects in the *eIF4E* gene family.

## Introduction

*Potato virus Y* (PVY) has been classified in the top 10 of the most economically and scientifically important plant viruses (Scholthof *et al.*, 2011). It is the type member of the genus *Potyvirus* in the *Potyviridae* family, one of the largest genera of plant viruses (Wylie *et al.*, 2017). PVY is transmitted by aphids and infects a large range of host plants worldwide, mostly within the *Solanaceae* family. In particular, it causes one of the most damaging diseases in cultivated tobacco (Quenouille *et al.*, 2013). PVY isolates are distributed into three biotypes, with the PVY^N^ and PVY^O^ biotypes separated by their ability or inability, respectively, to cause systemic veinal necrosis on tobacco (Moury, 2010; Singh *et al.*, 2008). Although mosaic symptoms impact less dramatically the yield and quality of tobacco crops, severe leaf necrosis induced by PVY^N^ isolates are a major concern for tobacco producers (Lacroix *et al.*, 2010; Rolland *et al.*, 2009; Tian *et al.*, 2011).

As for other potyviruses, the PVY genome consists of a single RNA molecule, polyadenylated at its 3’ end and covalently linked to a 25 kDa virus‐encoded VPg protein at its 5’ end (Leonard *et al.*, 2000; Revers and García, 2015). The completion of the viral cycle involves a complex interplay between virus‐ and host‐encoded factors, also called susceptibility factors. Absence or non‐adequacy of a single susceptibility factor can result in plant resistance, a phenomenon described as ‘loss‐of‐susceptibility’ (van Schie and Takken, 2014). Such recessive resistance genes against potyviruses have mainly been identified among translation initiation factors, including eukaryotic initiation factors 4E (eIF4E) and 4G (eIF4G) or their isoforms (Robaglia and Caranta, 2006; Sanfaçon, 2015; Truniger and Aranda, 2009; Wang and Krishnaswamy, 2012). Translation initiation factors 4E are essential components encoded by a small multigene family that bind to the mRNA cap structure at the 5’ end of most mRNAs (Browning and Bailey‐Serres, 2015). Natural resistance to potyviruses in crops such as lettuce (Nicaise *et al.*, 2003), pepper (Ruffel *et al.*, 2002), pea (Gao *et al.*, 2004) and tomato (Ruffel *et al.*, 2005) has been shown to result from non‐synonymous substitutions in *eIF4E* genes. Even if the exact mechanism(s) involved in *eIF4E*‐mediated resistance are still unclear (Wang and Krishnaswamy, 2012), it has been shown that the viral genome linked protein (VPg) can interact with eIF4E, mimicking the 5’ cap structure of messenger RNAs (mRNAs). In addition, potyviruses can evolve towards resistance breaking through the acquisition of mutations that either restore compatibility of their VPg with the host mutant eIF4E or allow an interaction with another member of the eIF4E family (Charron *et al.*, 2008; Gallois *et al.*, 2010, 2018; Lebaron *et al.*, 2016; Takakura *et al.*, 2018).

In tobacco, the main source of resistance against PVY is the *va* gene, which originates from the Virgin A Mutant (VAM) tobacco line obtained after X‐ray irradiation‐induced mutagenesis (Koelle, 1961; Singh *et al.*, 2008). A large deletion of almost 1 Mbp has been characterized in VAM using random amplified polymorphic DNA (RAPD) markers (Noguchi *et al.*, 1999) and a physical map of the corresponding region of chromosome 21 was recently developed (Dluge *et al.*, 2018). Julio *et al. *(2015) showed that a deletion of a particular copy of *elF4E* (hereafter named *eIF4E‐1*) encoded by the *S10760* gene is responsible for the *va* resistance. In particular, mutants obtained by ethyl methane sulfonate (EMS) mutagenesis with nonsense mutations in the *S10760* gene showed PVY resistance, confirming its direct involvement in *va* resistance (Julio *et al.*, 2015).

**Figure 1:**
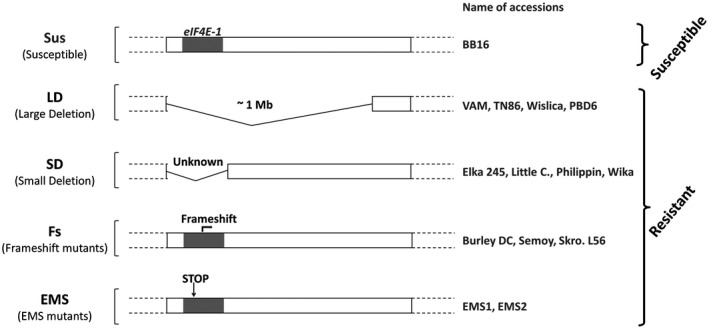
Type of mutation at the *eIF4E‐1* locus on chromosome 21 in the different *N. tabacum va* accessions. The *va* accessions listed on the right are classified into four categories (LD, SD, FS and EMS) according to the type of mutation at the *eIF4E‐1* locus. The EMS1 and EMS2 mutants correspond to the W50* and W53* mutants described in Julio et al. (2015). ‘Unknown’ indicates that the length of the deletion in the SD genotypes is not known.

However, the resistance of *eIF4E‐1 EMS*‐*KO* mutants is unstable, with the rapid and frequent emergence of PVY resistance‐breaking (RB) variants (Julio *et al.*, 2015). These observations suggest a possible role of the particular type of mutation affecting *eIF4E‐1* or of additional genetic factor(s) in the durability of the *va* resistance. Julio *et al. *(2015) identified 35 tobacco accessions carrying a *va* resistance allele in a germplasm collection. Depending on the type of mutation at the *va* locus, four groups of resistant genotypes were distinguished (Fig. 1). The first group, named LD for ‘large deletion’, corresponds to tobacco varieties carrying the 1 Mbp deletion on chromosome 21. The second group, named SD for ‘small deletion’, gathers tobacco varieties carrying a smaller deletion on chromosome 21 (as judged by the presence of markers absent in the LD group) that also results in a complete deletion of *eIF4E‐1*. The third category, named FS for ‘frameshift’ does not display any chromosomal deletion but a natural 2 bp deletion in the *eIF4E‐1* gene, leading to a C‐terminal truncated eIF4E‐1 protein of only 163 amino acids. The fourth group includes two EMS stop codons mutants, EMS1 and EMS2, that encode C‐terminal truncated eIF4E‐1 proteins of only 50 and 53 amino acids, respectively [corresponding to mutants W50* and W53* in Julio *et al. *(2015)].

The objectives of the present study were to better characterize the differences in resistance durability observed between tobacco genotypes differing in the *va* allele they possess. Through genetic and transcriptomic analyses we show here that resistance durability is strongly influenced by a complex genetic locus on chromosome 14, which contains other *eIF4E* copies. One of these copies is absent and replaced by a hybrid copy in the genotypes with the most stable resistance. In addition, the mRNA expression level of another copy is positively correlated with resistance durability. Taken together these results show that *va*‐mediated resistance durability can be explained by redundancy/competition effects between members of the *eIF4E* gene family, parallel to what was recently shown in tomato (Gauffier *et al.*, 2016).

## Results

### Phenotypic evaluation of the resistance conferred by different types of mutations at the *va* locus

**Figure 2:**
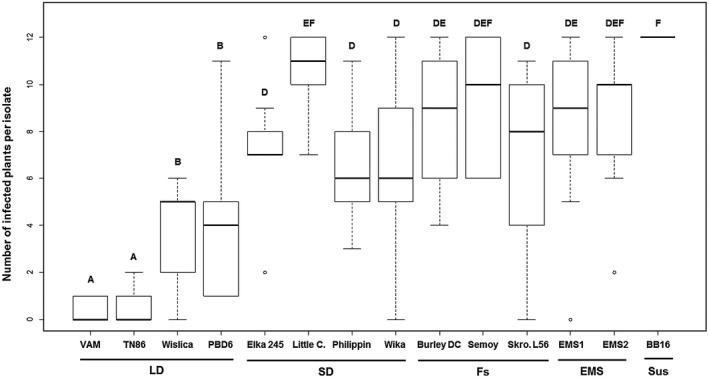
Boxplot representing the distribution of the numbers of infected plants per genotype at 30 dpi, taking into account the results obtained for the nine PVY isolates used. Each tobacco accession was challenged in two independent experiments by nine avirulent PVY isolates. Twelve plants were inoculated per isolate for each genotype. Viral accumulation was analysed at 30 dpi by ELISA. The values given are the average of the results obtained for each of the nine PVY isolates, for each of the two experiments. Multiple chi‐squared tests for pairwise comparisons were performed using the R software v. 3.2.5. Tobacco genotypes with same letter are not statistically different (*P* value > 0.05). Sus, susceptible BB16 genotype.

Our previous results showed that the resistance of *EMS‐KO eIF4E‐1* mutants is unstable, with the rapid and frequent emergence of PVY RB variants (Julio *et al.*, 2015). These observations suggest a possible role of the particular type of mutation affecting *eIF4E‐1* or of additional genetic factor(s) in the durability of the *va*‐mediated resistance. In order to investigate the potential role of the mutation type affecting *eIF4E‐1*, we challenged 13 accessions representing different classes of *va* mutations (Fig. 1) with nine PVY isolates avirulent on VAM and representative of the virus genetic diversity (five belonging to the N clade and four to the O clade, see the experimental procedures section). The susceptible BB16 genotype was used as a positive control for infection. For each of the 117 tobacco genotype–PVY isolate combinations, the infection rates (number of infected plants over number of inoculated plants) at 15 and 30 days post inoculation (dpi) are detailed in Table S1. Based on these results, Fig. 2 presents the distribution of the numbers of infected plants per genotype at 30 dpi globally for the nine PVY isolates used. On the BB16 susceptible control, systemic viral accumulation was detected in 94% of inoculated plants at 15 dpi and in 100% of plants at 30 dpi, regardless of the PVY isolate used (Table S1). On the opposite, the resistant LD VAM and TN86 accessions displayed the lowest proportion of plants showing PVY accumulation. Indeed, at 15 dpi PVY accumulation could not be detected in a single inoculated plant, while at 30 dpi only 2% and 5% of the plants showed detectable PVY accumulation for VAM and TN86, respectively (Table S1). The two other varieties with the large deletion, Wislica and PBD6, displayed a slightly higher proportion of plants with PVY accumulation. Accessions of the SD group displayed even higher frequencies of infected plants (Fig. 2), with a median between six and seven plants, except for the variety Little C (median of 11 plants). Finally, the groups showing the most variable results between PVY isolates and the highest frequency of infected plants correspond to the FS group (Burley DC, Semoy and Skro.L56 accessions) and the two EMS mutants (Table S1). Taken together, these results show that plants carrying a mutation affecting *eIF4E‐1* vary greatly in their behaviour towards PVY, ranging from very efficient resistance for LD genotypes to very ineffective resistance for FS and EMS genotypes (Fig. 2).

In order to confirm that within the LD group, VAM and TN86 display a higher resistance efficiency than Wislica and PBD6, a larger number of plants of the four LD genotypes were challenged in three independent experiments by two PVY isolates, SN3 and O139 (selected because they showed the highest infection rate in all challenged tobacco accessions). The EMS1 mutant and the susceptible BB16 genotype were used as controls. Table 1 shows that at 15 dpi, the four LD genotypes displayed significantly higher resistance efficiency than EMS1 and BB16, with 0% to 32% of infection for the LD genotypes, compared to 57% to 93% of infection for EMS1 and BB16, respectively (Table 1). At 30 dpi, there was no significant difference in the infection rates on PBD6 and EMS1 genotypes for the two PVY isolates, varying from 46% to 78%. The VAM genotype displayed significantly lower percentages of infection (1–2%) than the other LD genotypes (Table 1). Depending on the isolate, TN86 and Wislica displayed a significantly higher level of resistance than PDB6, confirming our previous observations showing that the efficiency of the resistance differs between LD genotype, with the VAM genotype displaying a significantly lower percentage of infection (Fig. 2).

**Table 1:** Response of the four LD *va*‐genotypes, EMS1 mutant and susceptible BB16 tobacco to PVY isolates O139 and SN3.

PVY isolate	Experiment	Phenotype^†^											
		VAM		TN86		Wislica		PBD6		EMS1		BB16	
		15 dpi	30 dpi	15 dpi	30 dpi	15 dpi	30 dpi	15 dpi	30 dpi	15 dpi	30 dpi	15 dpi	30 dpi
PVY^O^‐O139	1	0/30	1/30	2/30	3/30	6/30	8/30	2/30	17/30	9/30	10/30	10/13	13/13
	2	0/30	0/30	3/30	16/30	15/30	19/30	16/30	29/30	30/30	30/30	15/15	15/15
	3	1/30	1/30	0/30	2/30	2/30	10/30	2/30	16/30	27/30	30/30	15/15	15/15
	Infection rate (%)	1*^a^*	2*^A^*	6*^a^*	23*^B^*	26*^b^*	41*^B^*	22*^b^*	69*^C^*	73*^c^*	78*^C^*	93*^c^*	100*^D^*
PVY^O^‐SN3	1	0/30	0/30	0/30	0/30	1/30	1/30	1/30	2/30	0/30	0/30	10/13	10/13
	2	0/30	0/30	7/30	17/30	22/30	24/30	22/30	25/30	30/30	30/30	15/15	15/15
	3	0/30	1/30	0/30	3/30	3/30	8/30	6/30	14/30	21/30	30/30	15/15	15/15
	Infection rate (%)	0*^aʹ^*	1*^Aʹ^*	8*^aʹ^*	22*^Bʹ^*	29*^bʹ^*	37*^BʹCʹ^*	32*^bʹ^*	46*^CʹDʹ^*	57*^cʹ^*	67*^Dʹ^*	93*^dʹ^*	93*^Eʹ^*

^†^ Number of infected plants/number of inoculated plants for each independent experiment (1, 2 or 3). Infection was analysed by ELISA at 15 and 30 dpi. Multiple chi‐squared tests for pairwise comparisons were performed using the R software v. 3.2.5. For each PVY isolate, the infection rates labelled with the same letter (lower case or upper case for infection rates observed at 15 or 30 dpi, respectively) are statistically identical.

We challenged one tobacco genotype representative of each group (VAM for LD, Elka 245 for SD, Skro.L56 for FS and EMS1) with a larger collection of PVY isolates representative of the O and C clades. Table S2 shows that overall the resistance effectiveness is still statistically higher in VAM than in the other tested genotypes, with only nine infected plants out of 192 inoculated plants (5%) when taking into consideration all PVY isolates. The three other tested tobacco accessions, representative of the LD, FS and EMS mutant groups provided very similar results, with overall infection rates of 83–88%, which are not statistically different (Table S2). As before, 100% infection was recorded in BB16 used as a susceptible control.

### Infection of *va* tobacco genotypes is associated with the emergence of RB variants

Previously, Masuta *et al. *(1999) and Lacroix *et al. *(2011) identified amino acid changes in the central part of the VPg of PVY that were associated with the ability to overcome the *va* resistance. In the present study, we suspected that the varying number of infected plants, increasing with time of infection, corresponds to the emergence of RB variants. To confirm this hypothesis, the nucleotide sequence of the region encoding the central portion of the VPg was determined for a number of infected plants representing the vast majority of the viral isolate/tobacco genotype combinations tested. In total, the sequence of 91 independent viral progenies was determined for PVY^N^ isolates propagated in *va* tobacco accessions (Tables 2 and S3) and for 90 PVY^O^ progenies (Tables 2 and S4). The sequences obtained were then compared with the sequence of the corresponding inoculum. In the susceptible BB16 control accession, no mutation was observed in the 40 progenies analysed, irrespective of the PVY isolate (Tables S3 and S4). On the *va* accessions, a very different situation was observed. For the five PVY isolates belonging to the N clade, mutations in the central portion of the VPg were observed in all progenies, with amino acid substitutions affecting the lysine (K) at position 105 observed in 66 of the 91 progenies (72%, Tables 2 and S3). This position has previously been identified as a major RB determinant (Janzac *et al.*, 2014; Masuta *et al.*, 1999). In addition to VPg residue 105, point mutations at positions 101, 108, 109 and 119 were also observed, alone or in combination and always at much lower rates (Table 2 and Table S3). Positions 101, 108 and 109 have also been previously reported as being associated with *va* resistance breaking (Janzac *et al.*, 2014; Lacroix *et al.*, 2011). For the four PVY^O^ isolates, a total of 65 out of 90 analysed progenies (72%, Tables 2 and S4) displayed mutations in the central portion of the VPg, with again mutations at residue 105 being by far the most frequent (64%, Table 2). A few viral variants were also observed with mutations affecting VPg residues 101, 119, 120 and 121 (Tables 2 and S4). However, despite the presence of symptoms and detectable viral accumulation, no VPg mutation was observed in 28% of the PVY^O^ progenies coming from nine different *va* genotypes, and in particular in four EMS2 mutant progenies (Tables 2 and S4). On further propagation on EMS2 plants of two of these first passage progenies (derived, respectively, from the SN3 and LA4 isolates) a high infection rate was noted (92%) and a mutation at position 105 of the VPg was detected in these second passage progenies, suggesting that RB variants were probably already present, although in low proportion, in the first passage progenies. Altogether, these results indicate that the PVY infections observed on the different *va* tobacco accessions correspond to the emergence of RB variants having mutations at already identified key positions in the central region of the VPg. As we observed a high differential in RB frequency between tobacco accessions with the PVY‐O139 isolate, it was chosen to evaluate the durability of the *va* resistance in later experiments

**Table 2:** Amino acid substitutions identified in the VPg central region (amino acids 101–123) of the progenies of PVY^N^ and PVY^O ^isolates after passaging in the 13 different *va* tobacco accessions.

Amino acid change	Frequency	
	PVY^N^ variants	PVY^O^ variants
K105E, T, Q , M	73% (66/91)	64% (58/90)
K105T + G119H	–	1% (1/90)
S101G	24% (22/91)	4% (4/90)
S101G + V108I	1% (1/91)	–
V108I + G119C	1% (1/91)	–
E109D	1% (1/91)	–
S120R	–	1% (1/90)
N121Y	–	1% (1/90)
No mutation identified	–	28% (25/90)
TOTAL	91	90

The amino acid positions are numbered according to the VPg sequence of PVY‐N605 (GenBank X97895) and PVY‐O (GenBank U09509) for PVY^N^ and PVY^O^ isolates, respectively. Single and double mutations are listed. The nomenclature ‘K105E’ indicates the substitution of a lysine (K) by a glutamic acid (E) at position 105. For single mutations at position 105, the four substitutions observed (K105E, K105T, K105Q or K105M) are regrouped. C, cysteine; D, aspartic acid; E, glutamic acid; G, glycine; H, histidine; I, isoleucine; K, lysine; M, methionine; N, asparagine; Q, glutamine; R, arginine; S, serine; T, threonine; V, valine; Y, tyrosine. –, no mutation detected in the progenies at this position.

### A hybrid eIF4E‐^2‐3^ gene is present at chromosome 14 in the three LD genotypes showing the highest va‐mediated resistance durability

**Figure 3:**
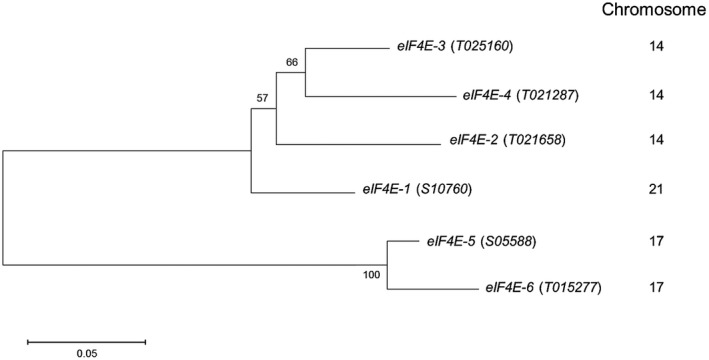
Phylogenetic analysis of the eIF4E protein family in *N. tabacum*. The phylogenetic tree was obtained using the maximum likelihood method from the alignment of amino acid sequences of eukaryotic initiation factor 4E proteins using the MEGA6 software with default parameters. The sequences used are as follows: *eIF4E‐1* (S10760, GenBank KF155696), *eIF4E‐2* (T021658, GenBank KM202068), *eIF4E‐3* (T025160, GenBank KM202070), *eIF4E‐4* (T021287, GenBank KM202069), *eIF4E‐5* (S05588, GenBank KM202071), *eIF4E‐6* (T015277, GenBank KM202067). Bootstrap values are shown on the branches and the branch length is proportional to the number of substitutions per site (sps).

We believe that the differences in *va* durability could be associated with genetic background effects and redundancy between *eIF4E* family members. We therefore decided to analyse the *eIF4E* family members to see if they might correlate with resistance durability. Tobacco is an allotetraploid derived from the diploids *N. sylvestris* and *N. tomentosiformis,* which are, respectively, susceptible and resistant to PVY. Sequences annotated as *eIF4E* or its isoforms *eIFiso4E* and novel cap‐binding protein (*nCBP*) have been identified in the transcriptomes of the two parental genomes and of tobacco (Julio *et al.*, 2015). Among the six *eIF4E* copies, four are derived from the *N. tomentosiformis* (T) genome, T015277, T021658, T021287, and T025160, and two from the *N.* s*ylvestris* (S) genome, S10760 and S05588. In the present study, *eIF4E‐1* stands for the *S10760* gene (*va* locus) and *eIF4E‐2, ‐3, ‐4, ‐5* and ‐*6* for the *T021658, T025160*, *T021287*, *S05588* and *T015277* genes, respectively (Fig. 3). The Blast function available on the Sol Genomics Network server (https://solgenomics.net/) (Edwards *et al.*, 2017) showed a localization of the *eIF4E‐2, 3 and 4* genes on chromosome 14 of the tobacco genotype K326 (PI552505), starting, respectively, at positions 109418388, 109399608 and 109292645, indicating these genes are located within a ca. 132 kbp region (Fig. S1). The short distance between *eIF4E‐2* and *eIF4E‐3* was confirmed using simple sequence repeat (SSR) markers (Bindler *et al.*, 2011) (see experimental procedures). Therefore, all three genes can be considered as a single large locus on chromosome 14. The two other *eIF4E‐5* and *eIF4E‐6* copies map on chromosome 17 (positions 79984931 and 70652013, respectively). Chromosome 17 may have undergone rearrangements between the *N. tomentosiformis* and *N. sylvestris* genomes (Edwards *et al.*, 2017), which may explain the simultaneous presence of *eIF4E‐5* gene (*S05588,* S‐genome origin) and *eIF4E‐6* (*T015277,* T‐genome origin).

The analysis of RNA‐seq data (Julio *et al.*, 2015) failed to identify amino acid polymorphisms in any of the six eIF4E copies between accessions with durable or unstable *va* resistance (Fig. S2), except for *eIF4E*‐3 in the LD genotypes. Indeed, only a partial coverage of the *eIF4E*‐3 gene was observed when mapping reads from the VAM, Start and Sk70 LD accessions, while complete coverage was observed for the other LD accessions (TN86, Wislica and PBD6) in the EMS1 mutant and in BB16 (Fig. S3).

**Figure 4:**
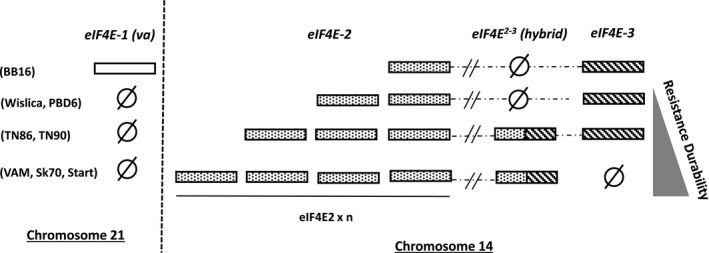
The different *eIF4E* copies in the seven LD accessions and the BB16 genotype on chromosomes 14 and 21. Ø indicates the absence of this genomic region. ≠ indicates that the drawing is not to scale. ‘eIF4E2 × n’ indicates that the *eIF4E2* gene is present in *n* copies. The *n* value is based on a relative quantitation estimated by TaqMan^®^ Copy Number Assay and varies between genotypes.

**Figure 5:**
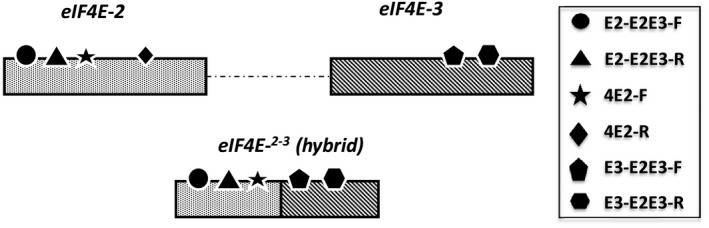
The position of the primers used for evaluation of copy number and identification of the hybrid copy on the coding sequence of *eIF4E‐2*, *eIF4E‐3* and *eIF4E‐* ^2‐3^ alleles present on chromosome 14.

A specific amplification of *eIF4E‐3* with the forward primer T025160F6 (Table S5) designed in the seemingly 5’ missing region in VAM and the reverse primer T025160R6‐2 (expressed region) was performed on the genomic DNA of a panel of tobacco accessions. Compared to other accessions (i.e. TN86, Wislica, PBD6, TN90, EMS1 mutant and BB16) from which the expected polymerase chain reaction (PCR) product of 324 bp was readily amplified, no amplification product was observed in VAM, Start and Sk70, confirming the existence of a genomic deletion affecting the 5’ region of *eIF4E‐3* in these accessions. This was further confirmed by high‐throughput sequencing of the VAM genomic DNA, which revealed instead the presence of a hybrid *eIF4E* copy, with the 5’ part of *eIF4E‐2* fused to the 3’ part of *eIF4E‐3*, hereafter named *eIF4E‐^2‐3^* (Fig. S4). This peculiar gene structure was confirmed by PCR in the VAM, Sk70 and Start genotypes (Fig. 4) using the various primer pairs shown in Fig. 5. Surprisingly, two other LD accessions, TN86 and TN90, were shown to contain both *eIF4E‐^2‐3^* and an entire *eIF4E‐3* gene (Fig. 4). Finally, we evaluated the copy number of *eIF4E‐2*, *eIF4E‐3* and *eIF4E‐^2‐3^* genomic DNA targets, using the TaqMan^®^ Copy Number Assay. As shown in Fig. 4, the LD accessions VAM, Sk70 and Start which show the highest *va*‐mediated resistance durability also combine multiple copies of *eIF4E‐2* and an *eIF4E‐^2‐3^* hybrid copy. Altogether, those results confirm the presence of a complex *eIF4E* locus on chromosome 14 (hereafter named *Chr14‐eIF4E*) differing between durable LD and non‐durable LD accessions.

### Genetic analysis indicates that *va *durability is influenced by the type of mutation at the *eIF4E‐1* locus and by the complex *Chr14‐eIF4E* locus

We first checked whether the *eIF4E‐^2‐3^* allele at the complex *Chr14‐eIF4E* locus co‐segregates with resistance durability. The F1 progeny of a cross between VAM and EMS1 showed a 12% infection rate at 30 dpi, comparable to that of VAM (Table 3), showing that the resistance durability trait is dominant. The phenotyping of 256 F2 progeny plants confirmed that the *eIF4E‐^2‐3^* allele co‐segregates with resistance durability. Using the same F2 progeny, we also checked whether the type of mutation at the *eIF4E‐1* locus [large deletion (*eIF4E‐1^LD^*) or point mutation (*eIF4E‐1^KO^*)] influences resistance durability. Indeed, homozygous *eIF4E‐1^LD^/eIF4E‐1^LD^* and heterozygous *eIF4E‐1^LD^/eIF4E‐1^KO^* F2 plants display infection rates comparable those of VAM and F1 hybrids, respectively (Table S6). Interestingly, the homozygous *eIF4E‐1^KO^/eIF4E‐1^KO^* F2 progenies display a significantly higher level of resistance durability than the EMS1 parent, with infection rates of 47% and 98%, respectively. This difference may reflect the influence of the locus *Chr14‐eIF4E*.

**Table 3:** The 5’ deletion of *eIF4E‐3* gene at the locus *eIF4E‐^2‐3^* on chromosome 14 co‐segregates with the resistance durability character.

	Genotype		Phenotype	*P *value (*chi^2^*)
	*eIF4E‐1 locus*	*eIF4E‐3 locus*		
VAM	*eIF4E‐1^LD^*/*eIF4E‐1^LD^*	*eIF4E‐^2‐3^*/ *eIF4E‐^2‐3^*	6% (2/33)	
EMS1	*eIF4E‐1^KO^*/*eIF4E‐1^KO^*	*eIF4E‐3/eIF4E‐3*	98% (32/33)	
F1	*eIF4E‐1^LD^*/*eIF4E‐1^KO^*	*eIF4E‐3/ eIF4E‐^2‐3^*	12% (4/33)	0.16^$^
F2	*eIF4E‐1^LD^*/*eIF4E‐1^LD^*	*eIF4E‐^2‐3^*/ *eIF4E‐^2‐3^*	5% (3/59)	1^†^
	*eIF4E‐1^KO^*/*eIF4E‐1^KO^*			
	*eIF4E‐1^LD^*/*eIF4E‐1^KO^*			
	*eIF4E‐1^LD^*/*eIF4E‐1^LD^*	eIF4E‐3/eIF4E‐3	100% (17/17)	1^‡^
	*eIF4E‐1^KO^*/*eIF4E‐1^KO^*			
	*eIF4E‐1^LD^*/*eIF4E‐1^KO^*			
	*eIF4E‐1^LD^*/*eIF4E‐1^LD^*	eIF4E‐3/ *eIF4E‐^2‐3^*	22% (40/180)	0.31^§^
	*eIF4E‐1^KO^*/*eIF4E‐1^KO^*			
	*eIF4E‐1^LD^*/*eIF4E‐1^KO^*			

The allele *eIF4E‐^2‐3^* corresponds to the hybrid form *eIF4E‐^2‐3^* on chromosome 14 as present in the VAM accession. The allele *eIF4E‐3* corresponds to the complete *eIF4E‐3* gene seen in most tobacco accessions. The allele *eIF4E‐1^LD^* corresponds to the large deletion on chromosome 21, which results in the complete deletion of the *eIF4E‐1* gene as seen in the VAM accession. The *eIF4E‐1^KO^* allele corresponds to the point mutation seen in the EMS1 mutant and leads to a C‐terminally truncated eIF4E‐1 proteins of 50 aa. F2 population (256 plants) from the cross (VAM × EMS1) segregating for the *eIF4E‐^2‐3^* were inoculated with PVY‐O139. The phenotype corresponds to the infection ratio (number of infected plants/number of inoculated plants) estimated by ELISA at 30 dpi. χ^2^ statistic tests were used to investigate whether the resistance durability phenotype (estimated as the infection rates) differed between (^$^) F1 plants and the parental VAM, (^†^) F2 plants homozygous *eIF4E‐^2‐3^*/*eIF4E‐^2‐3^* and the parental VAM, (^‡^) F2 plants homozygous *eIF4E‐3*/*eIF4E‐3* and the parental EMS1, (^§^) F2 plants heterozygous *eIF4E‐^2‐3^*/*eIF4E‐3* and the F1 progeny. The χ^2^ test was used to test the null hypothesis, stating that there is no significant difference between the expected and observed result. By statistical convention, we used the 0.05 probability level as our critical value. In all cases, the null hypothesis was accepted as the *P* value is > 0.05.

### Overexpression of the *eIF4E‐2* copy in the LD genotypes correlates with a higher durability of *va*‐mediated resistance

**Figure 6:**
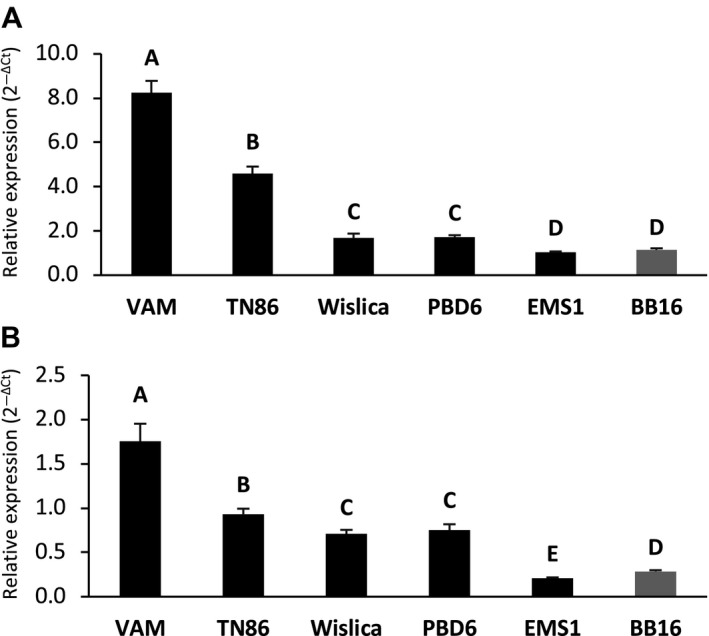
Relative quantification of *eIF4E‐2* gene expression by Q‐RT‐PCR. *EIF4E‐2* transcript levels were normalized to the expression of (A) *RL2* (*60S ribosomal protein L2*) or (B) *EF1‐α* (*elongation factor 1 alpha*) housekeeping genes, respectively. Gene expression was quantified in four pools of leaves sampled from six independent plants. The relative expression level (fold induction) was derived from the mean value of the four pools by comparing the two housekeeping genes to obtain the ΔCt (Ct*_eIF4E‐2_ – Ct_RL2 or EF1‐α_*) and was then calculated using the formula 2^–△Ct^ (as described by Schmittgen and Livak, 2008). The standard deviation between the four pools is indicated by vertical lines. Statistical analysis was performed using the Kruskal–Wallis rank sum test in R software v. 3.2.5. Significantly different values are noted with upper case when comparing the expression level of *eIF4E‐2* between genotypes. Tobacco genotypes labelled with the same letter are statistically identical (*P* value < 0.05). GenBank accessions numbers of the genes used: *eIF4E‐2* (KM202068), *RL2* (GenBank), *EF1‐α* (XM_009784954).

**Figure 7:**
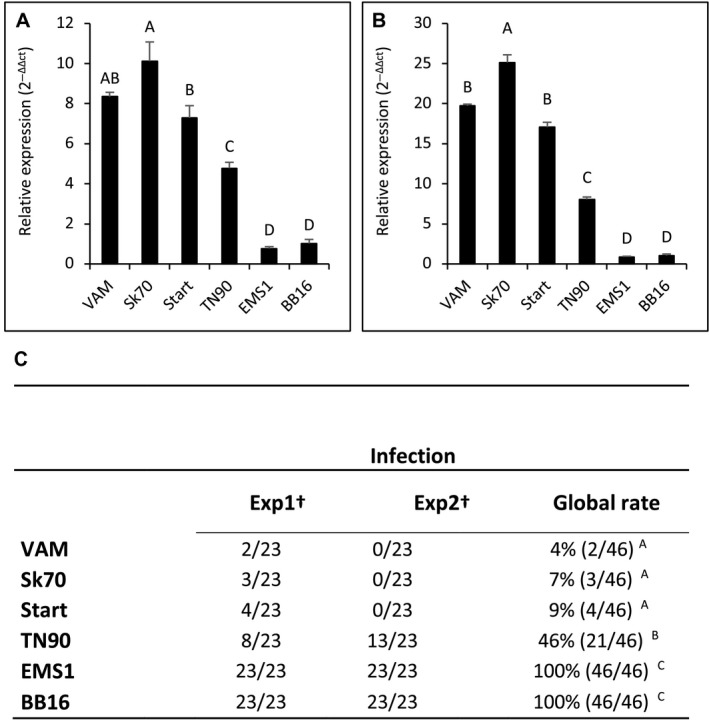
Relative quantification of *eIF4E‐2* gene expression by Q‐RT‐PCR and resistance level in various LD tobacco accessions. *EIF4E‐2* transcript levels were normalized to the expression of (A) *RL2* (60S ribosomal protein L2) and (B) *EF1‐α* (elongation factor 1 alpha) housekeeping genes, respectively. Gene expression was quantified from two pools of six independent plants. The relative expression level (fold induction) derived from the mean of the two pools by comparing the two housekeeping genes to obtain ΔCt (Ct*_eIF4E‐2_ –* Ct*_RL2 or EF1‐α_*) and BB16 (susceptible reference) value was used as the calibrator to determine the ΔΔCt and then calculated using the formula 2^–ΔΔCt^ (as described by Schmittgen and Livak, 2008). The standard deviation between the two pools is indicated by vertical lines. Statistical analysis was performed using the Kruskal–Wallis rank sum test in R software v. 3.2.5. Significantly different values are noted with capital letters (*P* value ≤ 0.05) when comparing the expression level of *eIF4E‐2 g*ene between each genotype. GenBank accession numbers of the genes used: *eIF4E‐2* (KM202068), *RL2* (X62500), *EF1‐α* (XM_009784954). (C) The different tobacco genotypes were challenged with PVY‐O139 isolate. †Number of infected plants/number of inoculated plants at 30 dpi. Infection rates correspond to the total number of infected plants/number of inoculated plants for two experiments (statistical analysis was performed using the multi‐comparison chi‐squared test in R software v. 3.2.5. Infection rates labelled with the same letter are statistically identical (*P* value < 0.05).

An initial analysis of RNA‐seq data suggested that *eIF4E‐2* (*T021658*) was more highly expressed in the resistant VAM accession (Julio *et al.*, 2015), suggesting the possible existence of an expression compensation mechanism between *eIF4E* orthologs derived from the two parental genomes in the amphidiploid tobacco. The results of a more extensive RNA‐seq analysis on a range of tobacco accessions carrying different types of mutations affecting the *eIF4E‐1* gene are shown in Table 4. They show that for all seven accessions with the large genomic deletion a higher expression of *eIF4E‐2* is observed as compared to the other *va* genotypes. It is also worth noting that among the LD accessions, three (VAM, Sk70 and Start) show a particularly striking overexpression (Table 4). Real‐time PCR confirmed these RNA‐seq results, using two different housekeeping genes for normalization (Fig. 6). The expression level of the *eIF4E‐2* gene was systematically increased by a factor 4 to 7 as compared to BB16 in TN86 and VAM, respectively, and by a factor of 1.5 in Wislica and PBD6. In contrast, there was no significant overexpression in the EMS1 mutant (Fig. 6A). The same trend was observed using another housekeeping gene (*EF1‐α*) as reference (Fig. 6B). Overall, both RNA‐seq data and quantitative reverse transcriptase‐polymerase chain reaction (Q‐RT‐PCR) indicate that *eIF4E‐2* overexpression is significantly stronger in VAM and TN86 as compared to Wislica and PBD6. The high overexpression level detected by RNA‐seq for Start and Sk70 was also confirmed by real‐time PCR, while TN90 showed an intermediate behaviour (Fig. 7A,B). The RNA‐seq data show the FS mutants and the small deletion accessions to behave similarly to EMS1 and BB16 in this respect. Taken together, these results demonstrate the existence of a very good correlation between resistance durability and the expression level of *eIF4E‐2*. This correlation is further reinforced by the observation that, similar to VAM, the Sk70 and Start accessions show the highest level of *eIF4E‐2* overexpression as well as a very high level of resistance durability when confronted with the PVY‐O139 isolate (Fig. 7C). Conversely, with an intermediate *eIF4E‐*2 overexpression level, TN90 displayed an intermediate resistance durability (Fig. 7C).

**Table 4:** RNASeq expression analysis of all *eIF4E* orthologs in the four groups of resistant accessions.

Genotype	Accession	*eIF4E‐1*	*eIF4E‐2*	*eIF4E‐3*	*eIF4E‐4*	*eIF4E‐5*	*eIF4E‐6*
Large deletion	VAM	0.4	173.1	4.0	4.9	16.9	20.0
	TN86	1.0	50.9	7.4	2.5	8.5	13.2
	Wislica	0.0	34.2	34.5	2.0	18.2	21.0
	PBD6	0.0	38.3	32.1	0.0	22.0	32.6
	Sk70	2.3	147.5	8.5	0.0	17.5	6.5
	Start	0.4	107.8	2.4	2.7	19.8	15.5
	TN90	0.0	78.5	18.5	1.9	15.4	12.7
Small deletion	Elka 245	0.4	11.3	11.8	2.1	14.2	15.6
	Little C.	0.0	19.2	13.4	1.2	16.8	14.9
	Philippin	0.0	17.6	13.0	1.2	22.6	14.6
	Wika	0.0	13.9	14.4	2.4	16.6	9.1
Frameshift	Burley DC	4.7	18.2	12.4	0.8	14.3	14.8
	Semoy	5.5	19.6	11.7	1.5	16.6	18.1
	Skro. L56	4.6	17.8	9.8	1.0	17.7	18.1
EMS mutant	EMS1	0.4	21.4	15.5	3.2	16.3	18.5

Normalized expression levels were obtained from RNA‐seq transcriptome analysis for uninfected tobacco accessions representative of the four categories of resistant plants (Julio *et al.*, 2015). Data were analysed with the ‘Set Up Experiment’ function and ‘two groups comparison’ by comparing characterized resistance to PVY (resistant versus susceptible). Data were normalized with the ‘Normalize’ function using default parameters (Method: Scaling; Values: Original Expression Value; Normalization value: Mean, CLC Genomics Workbench v5.5 software). Normalized data for each contig are shown.

## Discussion

### The durability of the resistance differs largely among *va*‐tobacco accessions

Here, we compared the durability of the resistance to PVY in 13 *va* tobacco accessions differing by their genetic background and the type of mutations affecting the *eIF4E‐1* gene (*va* locus), such as genomic deletions smaller than the large 1 Mbp found in VAM, natural frameshift mutations and EMS nonsense mutations. The 13 tobacco genotypes were challenged by a panel of PVY isolates representative of the virus diversity, and infection rates were monitored up to 30 days after inoculation to better analyse resistance breakdown. The PVY infections observed on the *va* genotypes correspond to the emergence of RB variants having, as previously shown, mutations in the VPg central region (Lacroix *et al.*, 2011; Masuta *et al.*, 1999; Moury *et al.*, 2004; Nicolas *et al.*, 1997). Our results highlight a variable resistance stability, with the LD accessions showing the most durable resistance, followed by the SD and FS accessions. The resistance of the EMS mutants was the least durable, confirming previous results (Julio *et al.*, 2015).

### Two genomic regions are involved in the durability of *va*‐mediated resistance in tobacco

Phenotypic, genetic and transcriptomic analyses showed that the type of mutation at the *eIF4E‐1* locus, together with the functionality and expression levels of other *eIF4E* copies at a complex locus on chromosome 14, impact *va*‐mediated resistance durability. The *eIF4E‐2, eIF4E‐3* and *eIF4E‐4* genes are all derived from the T genome and are the closest orthologs of the S genome *eIF4E‐1* (Fig. 3). The presence of these three closely related genes on chromosome 14 likely results from a triplication event in *N. tomentosiformis*, with some further chromosomal rearrangement(s) leading in LD accessions to the disruption of *eIF4E‐3*, in some cases to the appearance of *eIF4E‐^2‐3^* and to an increase in *eIF4E‐2* copy number.

Our observation that the resistance in VAM is significantly more durable than in most other *va* accessions confirms the results of Acosta‐Leal and Xiong (2008), who observed a rapid selection of RB variants in NC745, but not in VAM. They further suggested that functional components of the resistance were independently controlled by two segregating recessive genes in VAM: *va* itself (renamed *va1*) responsible for limiting potyvirus cell‐to‐cell movement and another locus, *va2,* limiting virus accumulation. They also suggested that *va1* and *va2* conditioned the stronger resistance of VAM (i.e. the resistance durability phenotype) and that the functional gene product of *va1*, *va2* or both loci, like most host factors interacting with VPg, might be an eIF4E factor. The fact that *va* encodes the eIF4E‐1 copy was demonstrated by Julio *et al. *(2015) and the present results strongly suggest that the *chr14‐eIF4E* complex locus could correspond to the *va2* locus, encoding gene products that may indeed interact with PVY VPg, as suggested by Acosta‐Leal and Xiong (2008).

### Redundancy among *eIF4E* isoforms might mediate *va* resistance durability

A compatible eIF4E isoform is required by potyviruses for their genome translation, replication, stabilization, and intercellular and systemic transport (Contreras‐Paredes *et al.*, 2013; Robaglia and Caranta, 2006; Sanfaçon, 2015; Wang and Krishnaswamy, 2012). Some potyviruses can also use several eIF4E isoforms in a given host (Gauffier *et al.*, 2016; Jenner *et al.*, 2010; Mazier *et al.*, 2011; Xu *et al.*, 2017). This suggests that in the absence of a functional eIF4E‐1 in *va*‐resistant genotypes, RB PVY variants may use another eIF4E isoform, similar to the situation when TuMV overcomes an *eIFiso4E* loss‐of‐function in Arabidopsis through VPg mutations allowing recruitment of eIF4E1 (Bastet *et al.*, 2018; Gallois *et al.*, 2018). However, our data suggest that the situation is even more complex in tobacco, and that besides the potential use of an eIF(iso)4E copy by RB PVY, as suggested by Takakura *et al *(2018), at least two other eIF4E copies may play a role in *va* resistance durability. Owing to their close proximity in the tobacco genome, however, it is not possible to genetically discriminate the potential contributions to durability of the overexpression of *eIF4E‐2* and the presence of the hybrid *eIF4E‐^2‐3^* gene (and/or lack of an integral *eIF4E‐3* copy).

Our results apparently diverge from the recent study of Takakura *et al. *(2018) showing that mutating the T‐genome *eIF(iso)4E* reduces the susceptibility to an RB PVY variant. However, these authors use an RB variant and cannot therefore evaluate how the tobacco genotype may affect the frequency with which such variants may appear, as was analysed in our study. The two series of results and conclusions are therefore in fact compatible and even complementary. It remains to be evaluated whether a mutation in *eIF(iso)4E* would impact the frequency of emergence of RB mutants.

Overall, a tight correlation network seems to link the superior durability seen in LD accessions such as VAM, Sk70 and START, the overexpression of *eIF4E‐2*, the presence of multi‐copies of *eIF4E‐2*, the presence of the *eIF4E‐^2‐3^* recombined gene and the deletion of *eIF4E‐1*. It is not currently possible to order in a cause and effect model these elements but, with its multiple levels, the durability/*eIF4E‐2* correlation appears to be the most promising. It should be noted that homeostatic control feedback mechanisms between eIF4E family members to compensate loss of function has been demonstrated in plants (Gallois *et al.*, 2018). This was previously reported in Arabidopsis, where eIF4E‐1 protein concentration increases to compensate for loss of eIFiso4E in the *KO‐eIF(iso)4E* (Duprat *et al.*, 2002). In tobacco cv Samsun, the depletion of *eIF(iso)4E* by an antisense down‐regulation strategy resulted in a compensatory increase in eIF4E protein levels (Combe *et al.*, 2005), while in *Brassica rapa*, overexpression of an *eIFiso4E* transgene induces the expression of endogenous *eIF4E* (Kim *et al.*, 2014), revealing another regulation mechanism. In tomato, it has also been suggested that protein–protein interactions between eIF4E‐1 and eIF4E‐2 lead to a yet unexplained eIF4E‐2 degradation (Gauffier *et al.*, 2016). In such a scenario, the deletion of *eIF4E‐1*, the loss of *eIF4E‐3* and the presence of *eIF4E‐^2‐3^* could all contribute, alone or in combination, to the overexpression of *eIF4E‐2*.

### The *eIF4E‐2* copy could act as a decoy increasing *va* resistance durability

**Figure 8:**
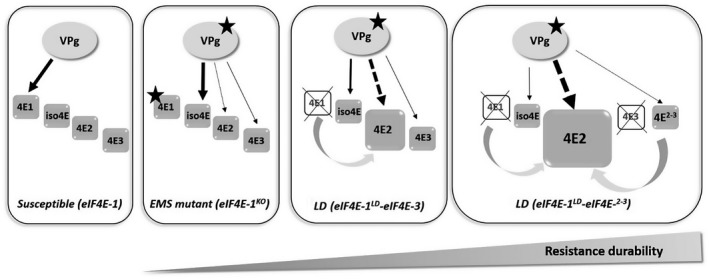
Model of eIF4E‐mediated *va* resistance durability in tobacco. In tobacco, the main eIF4E copy used by PVY is eIF4E‐1. In the resistant *va* accessions, either eIF4E‐1 is absent (crossed‐out) or a mutant C‐terminal truncated eIF4E‐1 protein is expressed (star). The appearance of RB variants, carrying mutations in the central region of the VPg (star), suggests that those RB variants can recruit another eIF4E factor for their infection cycle in the absence of the main susceptibility factor eIF4E‐1 or in the presence of a C‐terminal truncated protein. This factor can be eIF(iso)4E‐T or eIF4E‐2 or eIF4E‐3. The solid arrows represent productive recruitment for the viral infection whereas the dotted arrows represent unproductive recruitment. The resistance is more durable when eIF4E‐1 is absent (LD accessions). This could be explained by the overexpression of *eIF4E‐2* copy in the absence of eIF4E‐1, which is even exacerbated in the absence of eIF4E‐3 and presence of the hybrid eIF4E‐^2‐3^. The PVY or RB‐PVY variants could not use the eIF4E‐2 copy, which, when more abundant than other endogenous eIF4E copies, would monopolize translation initiation machinery. A surplus of eIF4E‐2 protein presumably makes ribosomal entry unavailable for the other endogenous eIF4E copy that may be used by the RB virus or could also monopolize the VPg in a nonfunctional interaction, limiting the possibility for the virus to evolve towards resistance breaking, leading to higher resistance durability.

In the scenario depicted in Fig. 8, PVY preferentially uses eIF4E‐1 to complete its infectious cycle in susceptible tobacco. In the resistant *va* accessions, either eIF4E‐1 is absent (LD and SD genotypes) or a non‐functional C‐terminally truncated eIF4E‐1 is expressed (FS and EMS genotypes). The absence of eIF4E‐1 or the failure of its truncated form to bind the viral VPg, a step that appears to be necessary although not sufficient to establish viral infection, could represent the key element in the *va*‐mediated resistance. However, the resistance is more durable in the LD genotypes because of the less frequent emergence of RB mutants. Assuming these RB mutants do not pre‐exist in the inoculum, their emergence requires a minimal level of replication, possibly involving imperfect interaction with (an)other isoform(s) such as eIF(iso)4E‐T (Takakura *et al*, 2018). One hypothesis to explain *va* durability is that PVY VPg could also interact in a non‐productive way with other isoforms, in particular with eIF4E‐2. The overexpression of *eIF4E‐2* observed in LD accessions and exacerbated in accessions lacking *eIF4E‐3* and carrying *eIF4E‐^2‐3^* could monopolize the VPg in a non‐functional interaction, limiting the possibility for the virus to evolve towards resistance breaking.

Consistent with this model, our results show that the *va* resistance durability trait is dominant and that eIF4E‐2 strongly differs from eIF4E‐1 in the loop I region close to the cap binding pocket (Fig. S4A), which contains amino acid sites known to be crucial for susceptibility to potyviruses (German‐Retana *et al.*, 2008; Robaglia and Caranta, 2006). All these elements support the model that overexpression of *eIF4E‐2* would act in a dominant negative manner (Chandler, 2003) similar to the resistance afforded through the transgenic overexpression of recessive resistance alleles in a susceptible background (Cavatorta *et al.*, 2011; Kang *et al.*, 2007). Other hypotheses are also possible, for example a direct involvement of the *eIF4E‐^2‐3^* copy, also acting as a decoy.

Despite its wide use, several adverse effects of the *va* gene have been observed. The resistant lines tend to have short and narrow leaves, and lower productivity (Noguchi *et al.*, 1999). This may reflect the presence of important genes in the large deletion containing the *va* gene. At the same time, this deletion may have negative effects on recombination during backcrosses and could impose a linkage drag, preventing restoration of a normal phenotype during breeding efforts (Yamamoto, 1992). Efforts have been developed to find new markers for background selection in backcross breeding programmes (Tajima *et al.*, 2002) but these have been incompletely successful in allowing the reduction of the negative effects of *va*. The second locus contributing to resistance durability, *chr14‐eIF4E,* could be transferred by marker‐assisted selection (MAS) in elite lines. A strategy to achieve a fully durable resistance could be to combine in the same plant a complete deletion of *eIF4E‐1* and *eIF(iso)4E‐T* with the *chr14‐eIF4E* locus. As new breeding techniques become also available for genome editing (Abdallah *et al.*, 2015; Andersen *et al.*, 2015), such approaches could be envisioned for introducing sequence‐specific deletions in the *eIF4E‐1* and *eIF(iso)4E‐T* genes in a *chr14‐eIF4E* background, opening new perspectives to improve our ability to manipulate and deploy durable tobacco resistance against one of its major viral pathogens, *Potato virus Y.*

## Experimental Procedures

### Plant material

The PVY‐susceptible *N. tabacum* cv. *Xanthi* was used to produce viral inoculum for biological characterization experiments. The 163 accessions used for RNA‐seq analysis belong to the Imperial Tobacco LTD collection and were described in Julio *et al.* (2015) as well as the *eIF4E‐1* EMS stop codons mutants EMS1 and EMS2 corresponding to W50* and W53* mutations (with tryptophan amino acid at position 50 or 53 in the sequence replaced by a stop codon). Another *eIF4E‐2* mutant (named E3‐762) was obtained from the EMS collection described in Julio *et al.* (2015). Among the tobacco accessions of known PVY resistance status previously characterized using *S10760*‐linked markers as well as other markers associated with the deletion affecting the *va* genomic region, the VAM, TN86, Wislica, PBD6, START, SK70 and TN90 accessions display a *va*‐resistance allele associated with a large deletion on chromosome 21 (Julio *et al.*, 2015). In this paper, those accessions belong to the LD group (Fig. 1). In two dark air‐cured (Little C. and Philippin) and two flue‐cured (Wika and Elka 245) tobaccos, the *S10760* (*eIF4E‐1*) gene could not be amplified, which suggests that it is either deleted or mutated in a way that prevents its amplification (Julio *et al.*, 2015). However, the other markers linked to *va* are not deleted in these plants, which suggests that they carry a smaller form of the deletion affecting *va*. Those accessions are therefore classified in this study in the SD group. The last group of accessions concerns resistant plants in which the *S10760 eIF4E‐1* gene is present and which do not appear to have a deletion on chromosome 21. However, a 2‐bp deletion in the *S10760* gene has been identified in two dark air‐cured varietal types (Semoy and Skro. L56) and in one burley type (Burley DC), which would truncate the encoded protein and explain the observed resistance, as for the two EMS mutants analysed in Julio *et al.* (2015). In this study, those three accessions are classified into the FS group (Fig. 1). Healthy and infected plants were maintained in a separate insect‐proof greenhouse compartment (18⁄25 °C night⁄day) or in a climate chamber (18/20 °C, 8 hours night/16 hours day).

### Viral material

The PVY isolates used to challenge the different tobacco accessions belong to the clade N (CSA1, LA7, CSA6, MaSan4, 1108) and the clade O (O139, LA4, SN3 and SAV8), depending on their ability to induce (N) or not (O) systemic veinal necrosis symptoms in tobacco (Jakab *et al.*, 1997; Moury, 2010; Singh and Singh, 1996) (Table S1). Those isolates were selected according to their pathogenic and biological properties in tobacco after a field survey carried out in tobacco plots in France in 2007 (Janzac *et al.*, 2014; Lacroix *et al.*, 2010). The other PVY isolates listed in Table S2 belong to the C clade, more specifically to the C1 (SON41, Alger1, Marti3, LYE84.2, CAA157, CAA141, CAA16, LYE72‐Puc2Pl3) or C2 (Cadgen, LYE90v) groups. Some isolates are PVY recombinants between O and C clades (CAA156, LYE245), others belong to the groups PVY^NTN^ (NTN‐H) and PVY^Wi^ (WilgaP) (Glais *et al.*, 2002). PVY isolates belonging to the N clade are Pologne 6 puc3 pl2 and N605, and one isolate comes from Brazil, Bresil 1054. Some of the isolates are referenced in (Ben Khalifa *et al.*, 2012; Moury, 2010; Moury *et al.*, 2004, 2011; Woloshuk *et al.*, 1993).

### Virus inoculation and detection

Each PVY isolate was first propagated into *N. tabacum* cv *Xanthi* plants in order to prepare viral inoculum. Fifteen days after inoculation, the systemically infected apical leaves (~12 g) were ground in liquid nitrogen and the powder stored at ‐80 °C. Total RNAs were extracted from 1 mg of powder, using the SV Total RNA Isolation System (Promega, Madison, USA) extraction kit. The viral RNAs were then quantified by real‐time PCR on a light cycler 480 (Roche, Bâle, Switzerland) using the AgPath‐ID Kit™. For each PVY isolate, the viral inoculum was then calibrated at a concentration of 10^8^ copies of viral RNA/100 µL in the inoculation buffer (50 mM Na_2_HPO_4,_ 50 mM KH_2_PO_4_, supplemented with 40 mM sodium diethyldithiocarbamate, pH = 7.2).

Tobacco plants with two fully expanded true leaves were inoculated manually approximately 2 weeks after sowing. The two lastly developed leaves were inoculated with 100 µL of the calibrated viral inoculum supplemented with carborundum. Evaluation of PVY infection in non‐inoculated leaves was performed by DAS enzyme‐linked immunosorbent assay (ELISA) (Clark and Adams, 1977; Lacroix *et al.*, 2010) at 15 and 30 dpi. Absorbance values at 405 nm (*A*_405_) with the background subtraction of buffer samples were considered for analysis. Samples were considered positive when their *A*_405_ was higher than three times the mean *A*_405_ of non‐inoculated samples. Polyclonal antibodies detecting all PVY isolates, monoclonal antibodies specific for PVY^N^ isolates (kindly provided by Maryse Guillet, INRA‐FN3PT, Le Rheu, France) and monoclonal antibodies specific for PVY^O/C^ isolates (Neogen Ltd, Lansing, Michigan, USA) were used.

### Analysis of VPg sequence in the viral progenies

The viral progenies present in infected plants were amplified by immuno‐capture RT‐PCR (IC‐RT‐PCR) as described previously (Glais *et al.*, 1998) using the 3′NTR‐reverse primer for the production of cDNA and primer pair RJ2‐F and NIa‐R (Table S5) to amplify the VPg region. Thermal cycling conditions were as follows: 2 min at 95 °C followed by 40 cycles of 1 min at 94 °C, 1 min at 57 °C and 1 min at 72 °C, followed by an elongation step of 10 min at 72 °C. Each PCR product was sent to Genoscreen (Lille, France) for sequencing.

### Analysis of *eIF4E‐2* gene expression by quantitative RT‐PCR

Plant samples were ground with a mixer mill (Retsch, Haan, Germany) at –80 °C and total RNAs were isolated using the NucleoSpin^®^ RNA plant Kit (Macherey Nagel, Düren, Germany). The RNAs obtained were treated with TURBO DNA‐free^TM^ Kit (Invitrogen, Thermo Fischer Scientific, Waltham, Massachusetts, USA) and the concentration and purity were determined by measuring absorbance at 230, 260 and 280 nm in a microplate UV‐Vis spectrophotometer (EPOCH^TM^ BioTek instrument, Winooski, Vermont, USA). The total RNA was adjusted to 50 mg/mL and was reverse transcribed according to the manufacturer’s instructions using the RevertAid H Minus enzyme (Thermo Scientific) and oligo(dT) primers. The cDNA was used to perform the real‐time Q‐RT‐PCR on the Light Cycler 480 Instrument II (Roche, Bâle, Switzerland), using the Light Cycler^®^480 SYBR Green I MASTER Kit (Roche, Bâle, Switzerland) following the protocol described in Michel *et al.* (2018). The PCR mix per well included 10 µL of Master mix, 0.6 µL of each primer (0.3 mM) eIF4E‐2 Fwd and eIF4E‐2 Rev (Table S5), and 5 µL of cDNA. Thermal cycling conditions were as follows: 15 s at 95 °C followed by 40 cycles of 5 s at 95 °C, 20 s at 57 °C and 30 s at 72 °C. The average values were normalized to the expression of two reference genes: *RL2* (*60S ribosomal protein L2*, GenBank X62500) and *EF1‐α* (*elongation factor 1‐alpha*, GenBank XM_009784954). The relative expression level (fold induction) compared to BB16 genotype was calculated with the formula 2^–ΔΔCt ^(Schmittgen and Livak, 2008). Statistical analysis was performed using the Kruskal–Wallis rank sum test in R software v. 3.2.5.

### Mapping of *eIF4E‐2* and *eIF4E‐3* genes

An improved genome assembly covering 90% of predicted *N. tabacum* genome size was recently released on the Sol Genomics Network (Edwards *et al.*, 2017). In order to map the different copies of *eIF4E* genes, we used the Blast function available online (https://www.sgn.cornell.edu/). To confirm the position of both *eIF4E‐2* and *eIF4E‐3* genes, we used two F2 segregating populations and SSR markers available in this region of chromosome 14 (Bindler *et al.*, 2011) with the same protocol as described in Julio *et al.* (2015).

The *eIF4E‐3* mapping was confirmed using 190 F2 individuals from the VAM (hybrid *eIF4E‐^2‐3^*) x Wika cross. As we couldn’t find any polymorphism in *eIF4E‐2* in the collection of 163 varieties, we used an EMS mutant in *eIF4E‐2* (named E3‐762) to create a segregating population: mapping was performed on 90 F2 individuals resulting from the VAM x E3‐762 cross. Between 76 cM and 93 cM, markers PT51975, PT50395, PT60091, PT51004, PT60558 and PT53953 were tested on parents of both crosses. Only two markers were polymorphic on both crosses: PT60091 amplified a 172 bp fragment in VAM, a 157 bp fragment in Wika and a 220 bp fragment in the mutant E3‐762 while PT51004 amplified a 170 bp fragment in the mutant E3‐762, 170 bp in Wika and nothing in VAM (null allele). SSR band sizes were scored on an ABI3130xl Genetic Analyzer with POP‐7™ Polymer (Applied Biosystems, Foster City, California, USA) using GeneMapper^®^ v4.0. The *eIF4E‐2* and *eIF4E‐3* genes were amplified with the same primers as those used for EMS mutant detection (Table S5). Analysis of PCR products was performed with a CE‐SSCP protocol for *eIF4E‐2* PCR product and with the POP‐7™ Polymer for *eIF4E‐3* PCR products. These gene‐specific markers as well as the PT60091 and PT51004 SSR markers were tested on VAM x Wika and VAM x E3‐762 F2 progenies. This resulted in complete linkage between *eIF4E* genes, PT60091 and PT51004.

### Copy number analysis for *eIF4E‐2*, *eIF4E‐3* and *eIF4E‐^2‐3^*

In order to evaluate the copy number of genomic DNA targets for *eIF4E2, eIF4E3* and *eIF4E‐^2‐3^*, we used the TaqMan^®^ Copy Number Assay based on gold‐standard TaqMan MGB probe chemistry. TaqMan^®^ Copy Number Assays are run together with a TaqMan Copy Number Reference Assay in a duplex qPCR reaction; the copy number assay detects the target sequence and the reference assay detects a sequence that is known to be present in two copies in the diploid genome. Here, the reference sequence was the nitrate reductase gene (GenBank XM_016606716) for all copy number assays that were amplified with primers NR4‐F and NR4‐R and the probe NR4 (Table S5).

The 5ʹ region common to *eIF4E‐2* and *eIF4E‐^2‐3^* was amplified in a multiplex reaction with the primers pair E2‐E2E3‐F and E2‐E2E3‐R and the probe E2‐E2E3 (Fig. 6, Table S5). The region specific to *eIF4E‐2* was amplified in multiplex with the primer pair 4E2‐F and 4E2‐R and the probe 4E2 (Table S5). The 3ʹ region specific to *eIF4E‐3* was amplified in a multiplex reaction with the primer pair E3‐E2E3‐F and E3‐E2E3‐R and the probe E3‐E2E3 (Fig. 6, Table S5). All PCR reactions were carried out in a 10 µL volume containing 5 ng of DNA, 1x TaqPath™ ProAmp™Master Mix (Applied Biosystems™, Foster City, California, USA), 900 nM of each primer and 250 nM of each probe. The amplification was conducted using a QuantStudio‐3 Real‐Time PCR (Applied Biosystems, Foster City, California, USA) as follows: 95 °C for 10 min, 40 cycles of 95 °C for 15 s and 60 °C for 1 min. Results were analysed by the relative quantitation method using CopyCaller^®^ Software v2.0 (Applied Biosystems, Foster City, California, USA).

### Sequencing of tobacco VAM accession genomic DNA

VAM DNA was extracted independently from eight plantlets using a Qiagen Dneasy kit, checked for concentration and mixed together. Libraries were prepared with an Illumina TruSeq PCR‐free kit and sequenced on one lane of HiSeq 3000 (paired‐end 2 × 150 pb) generating 176 403 416 paired‐end reads. Data were imported in CLC Genomic Workbench, as well as the *N. tabacum* v1.0 reference genome (Edwards *et al*, 2017) available on the Solanaceae Genomics Network (SGN; https://solgenomics.net). The function ‘Map Read to Contigs’ was used. The parameters used were as default, with length fraction = 1 and similarity fraction = 0.98.

### Screening for mutation in the *eIF4E‐2* gene

A new EMS mutant collection was developed from seeds of the TN90 resistant variety as described in Julio *et al.* (2008) and used to detect *eIF4E‐2* mutants. Specific primers for *eIF4E‐2* gene were designed to amplify an exon region in each gene. Primers T021658E6TF and T021658E3T2R (Table S5) were used to amplify an amplicon of 329 bp of *eIF4E‐2*. PCR amplification was carried out with FAM and VIC dye‐labeled primer pairs in a 10‐μL volume containing 2 μL DNA, 0.05 U AmpliTaq^®^ Polymerase (Applied Biosystems, Foster City, California, USA), 1 μL 10 × AmpliTaq buffer, 0.5 μL dNTPs (Applied Biosystems, 2.5 mM each) and 7 ng of each primer. PCR was conducted using a thermal cycler (GeneAmp^®^ PCR System 9600, Applied Biosystems) as follows: 28 to 32 cycles of 94 °C for 30 s, 62 °C for 45 s and 72 °C for 1 min, followed by 7 min at 72 °C for final extension. *eIF4E‐2* PCR products were analysed by capillary electrophoresis‐single strand conformation polymorphism and sequenced as described in Julio *et al.* (2008).

## Supporting information


**Fig. S1** Position of the *eIF4E‐2, eIF4E‐3* and *eIF4E‐4 *genes on the *N. tabacum* (var. K326; PI552505) chromosome 14.

**Fig. S2** Amino acid sequence alignment of the various *eIF4E* proteins of *Nicotiana tabaccum*.

**Fig. S3** Mapping of the RNASeq reads against the *eIF4E‐3* consensus sequence for the LD accessions VAM, TN86, Wislica, PBD6, Sk 70 and Start, the EMS1 mutant and the susceptible BB16 accession.

**Fig. S4** Amino acid sequence polymorphism between eIF4E 1, eIF4E 2, eIF4E 3 and the hybrid eIF4E ^2‐3^ copy present in some LD genotypes of *Nicotiana tabaccum*.

**Table S1** Response of *va *tobacco genotypes to PVY isolates.

**Table S2** Response of representative *va *tobacco genotypes to various PVY isolates.

**Table S3** Amino acid changes in the VPg central region (amino acids 101 123) of the progenies of five PVY^N^ isolates, following infection of 13 different *va* tobacco accessions, in comparison with the sequence of the parental isolates.

**Table S4** Amino acid changes in the VPg central region (amino acids 101 123) of the progenies of four PVY^O^ isolates, following infection of 13 different *va* tobacco accessions, in comparison with sequence of the parental isolates.

**Table S5** List of primers and probes used in this study.

**Table S6** The deletion at the locus *eIF4E‐1^LD^* on chromosome 21 co‐segregates with the resistance durability character.
