# Supplementary material for: A complex eIF4E locus impacts the durability of va resistance to Potato virus Y in tobacco
Source: Mol Plant Pathol. 2019 May 21;20(8):1051–66. doi: 10.1111/mpp.12810 (PMC6640182; doi:10.1111/mpp.12810)
Supplement: Supplementary file 1 — Fig. S1 Position of the eIF4E‐2, eIF4E‐3 and eIF4E‐4 genes on the N. tabacum (var. K326; PI552505) chromosome 14. [file MPP-20-1051-s001.docx]

**
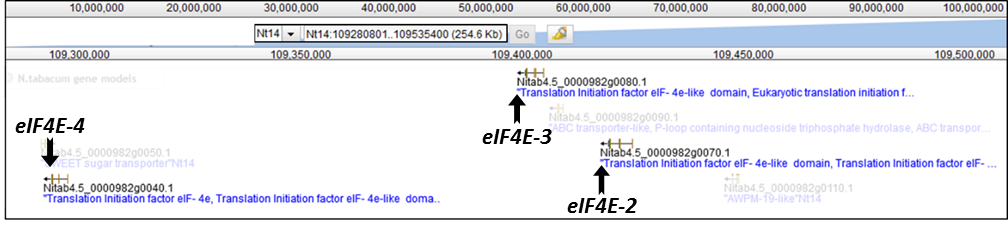
**

**Figure S1. Position of the *eIF4E-2, eIF4E-3, and eIF4E-4* genes on the *N. tabacum* (var. K326; PI552505)** **chromosome 14.** The figure shows the results of Blast analysis of the *eIF4E-2* (*T021658), eIF4E-3 (T025160)* and *eIF4E-4 (T021287)* genes on the *N. tabacum* reference genome available on the Sol Genomics Network server. These genes are annotated as translation initiation factors and positionned on chromosome 14 between positions 109292645 and 109424002 (132 Kb).
