# Supplementary material for: A complex eIF4E locus impacts the durability of va resistance to Potato virus Y in tobacco
Source: Mol Plant Pathol. 2019 May 21;20(8):1051–66. doi: 10.1111/mpp.12810 (PMC6640182; doi:10.1111/mpp.12810)
Supplement: Supplementary file 2 — Fig. S2 Amino acid sequence alignment of the various eIF4E proteins of Nicotiana tabaccum. [file MPP-20-1051-s002.docx]

....|....| ....|....| ....|....| ....|....|

10 20 30 40

**eIF4E-1 (S10760)**  MAEEAEK-LR VDEVEVADDG PEE-GEIVDE SDDTASYLG-

**eIF4E-2 (T021658)**  MAEESEK-LR VDEVEVADDG PEE-GEIVGE SDDTASYLS-

**eIF4E-3*(T025160)**  MAEESEK-LR VDEVEVADDG PEE-GEIVGE SDDTASYLS-

**eIF4E-4 (T021287)**  MAEESEK-LR VDEVEVADDG PEE-GEIVGE SDDTVSHLS-

**eIF4E-5 (S05588)**  MVDEVEKPVS LEESKTNTRE VEEEGEIVGE SDDTMSSLGN

**eIF4E-6 (T015277)**  MVDEVEKPAS LEESKTNTRE VEEGAEEVIE SDDTMSSLGN

**....|....| ....|....| ....|....| ....|....|**

50  **60 70**  80

**eIF4E-1 (S10760)** KEIKPKHPLE NSWTFWFDNP MAKSRQAAWG SSLRELYTFS

**eIF4E-2 (T021658)** KEIKAKHLLE NSWTFWYDK- NTKSRQAAWG SFLREVYTFS

**eIF4E-3 (T025160)** KEIKAKHPLE NSWTFWFDNP TAKSRQAGWG SSLRDVYTFS

**eIF4E-4 (T021287)** KEIKAKHPLE NSWTFWFDNP MAKSRQAAWG SSLRDVYTFS

**eIF4E-5 (S05588)** PSMAMKHALE HSWTFWFDNP SGKSKQAAWG SSIRPIYTFS

**eIF4E-6 (T015277)** PCKAMKHPLE HSWTFWFDNP SGKSKQAAWG SSIRPIYTFS

....|....| ....|....| ....|....| ....|....|

90 100 110 120

**eIF4E-1 (S10760)** TVEDFWGVYN NINHPSKLVV GADFHCFKHK IEPKWEDPVC

**eIF4E-2 (T021658)** TIEDFWGVYN NINHPSKLVT GADFHCFKHK IEPKWEDPVC

**eIF4E-3 (T025160)** TIEDFWGVYN NINHPSKLVS GADLHCFKHK IEPKWEDPVC

**eIF4E-4 (T021287)** TVEDFWGAYD NFQHPSKLVA GAGLYCFKHK IEPKWEDPIC

**eIF4E-5 (S05588)** TVEDFWSVYN NIHHPSKLAV GADFHCFKNK IEPKWEDPVC

**eIF4E-6 (T015277)** TVEDFYSVYN NIHHPSKLAV GADFHCFKNK IEPKWEDPVC

....|....| ....|....| ....|....| ....|....|

130 140 150 160

**eIF4E-1 (S10760)** ANGGNWTMSF S-KGKSDTSW LYTLLAMIGH QFDHGEEICG

**eIF4E-2 (T021658)** ANGGKWTMSF S-KGKSDTSW LYTLLAMIGH QFDHGDEICG

**eIF4E-3 (T025160)** ANGGKWTMSF S-KGKSDTSW LYTLLAMIGH QFDHGDEICG

**eIF4E-4 (T021287)** ANGGKWTMSF W-KGKSDTSW LYTLLAMIGH QFDHGDEICG

**eIF4E-5 (S05588)** ANGGKWTMSF S-RGKSDTCW LYTVLAMIGE QFDCGDEICG

**eIF4E-6 (T015277)** ASGGKWTMSF S-RGKSDTCW LYTVLAMIGE QFDCGDEICG

....|....| ....|....| ....|....| ....|....|

170 180 190 200

**eIF4E-1 (S10760)** AVVSVRN--- KGDKIALWTK NAANETAQVS IGKQWKEFLD

**eIF4E-2 (T021658)** AVVNVRG--- KEDKIALWTK NAANETAQVS IGKQWKEFLD

**eIF4E-3 (T025160)** AVVSVRAGRA KGEKIALWTK NAVNETAQVS IGKQWKEFLD

**eIF4E-4 (T021287)** AVVSIRH--- KGEKIALWTK NAADETAQVS IGKQWKEFLD

**eIF4E-5 (S05588)** AVINVRV--- RQEKIALWTR NAANETAQVS IGKQWKEFLD

**eIF4E-6 (T015277)** AVINVRV--- RQEKIALWTR NAANETAQVC IGKQWKEFLD

....|....| ....|....| ....|..

210 220

**eIF4E-1 (S10760)** YSNSIGFIFH DDSMRLGRGA KNRYTV.

**eIF4E-2 (T021658)** YSDSIDFIFH EDAERHGRGA KNRYTV.

**eIF4E-3 (T025160)** YSDSIGFIFH DDAKRLDKGA KNRYTV.

**eIF4E-4 (T021287)** YSDSIGFIVH DDAKRLGRGA KYRYTV.

**eIF4E-5 (S05588)** YNDSVGFIFH DDAKKLDRAA KNRYSV.

**eIF4E-6 (T015277)** YNDSIGFIFH DDAKKLDRAA KNRYSV.

**Figure S2. Amino acid sequence alignment of the various *eIF4E* proteins of *Nicotiana tabaccum*.** GenBank accession numbers: *eIF4E-1* (S10760, KF155696); *eIF4E-2* (T021658, KM202068); *eIF4E-3* (T025160, KM202070); *eIF4E-4* (T021287, KM202069); *eIF4E-5* (S05588, KM202071); *eIF4E-6* (T015277, KM202067). *The *eIF4E-3* sequence corresponds to the protein predicted from Edwards Reference genome (Edwards *et al.*, 2017).
