# Supplementary material for: A complex eIF4E locus impacts the durability of va resistance to Potato virus Y in tobacco
Source: Mol Plant Pathol. 2019 May 21;20(8):1051–66. doi: 10.1111/mpp.12810 (PMC6640182; doi:10.1111/mpp.12810)
Supplement: Supplementary file 3 — Fig. S3 Mapping of the RNASeq reads against the eIF4E‐3 consensus sequence for the LD accessions VAM, TN86, Wislica, PBD6, Sk 70 and Start, the EMS1 mutant and the susceptible BB16 accession. [file MPP-20-1051-s003.docx]

**
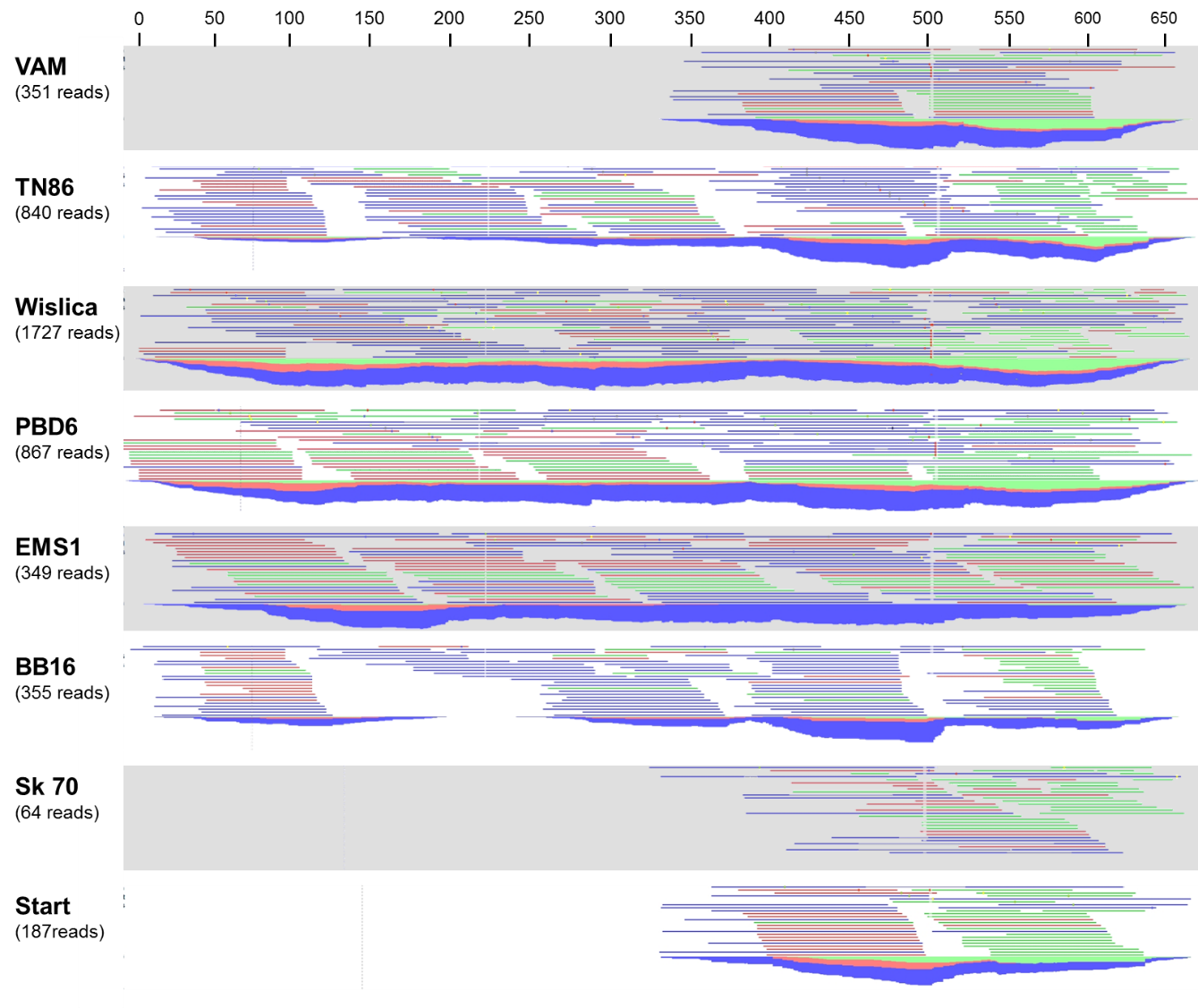
**

**Figure S3. Mapping of the RNASeq reads against the *eIF4E-3* consensus sequence for the ‘LD’ accessions VAM, TN86, Wislica, PBD6, Sk 70 and Start, the EMS1 mutant and the susceptible BB16 accession.** CLC-Genomic Workbench tracks are shown. Mapping reads are colored according to the following code: single reads mapping in their forward or reverse direction are respectively green or red. Paired reads are blue.
