# Supplementary material for: A complex eIF4E locus impacts the durability of va resistance to Potato virus Y in tobacco
Source: Mol Plant Pathol. 2019 May 21;20(8):1051–66. doi: 10.1111/mpp.12810 (PMC6640182; doi:10.1111/mpp.12810)
Supplement: Supplementary file 4 — Fig. S4 Amino acid sequence polymorphism between eIF4E 1, eIF4E 2, eIF4E 3 and the hybrid eIF4E 2‐3 copy present in some LD genotypes of Nicotiana tabaccum. [file MPP-20-1051-s004.docx]

**A**

**eIF4E-1** **(S10760, va)** MAEEAEKLRVDEVEVADDGPEEGEIVDESDDTASYLGKEIK

**eIF4E-2** **(T021658)** MAEESEKLRVDEVEVADDGPEEGEIVGESDDTASYLSKEIK

Loop I Loop II

**eIF4E-1** PKHPLENSWTFWFDNPMAKSRQAAWGSSLRELYTFSTVEDFWGVYNNINHPSKLVVGADF

**eIF4E-2** AKHLLENSWTFWYDK-NTKSRQAAWGSFLREVYTFSTIEDFWGVYNNINHPSKLVTGADF

**eIF4E-1** HCFKHKIEPKWEDPVCANGGNWTMSFSKGKSDTSWLYTLLAMIGHQFDHGEEICGAVVSV

**eIF4E-2** HCFKHKIEPKWEDPVCANGGKWTMSFSKGKSDTSWLYTLLAMIGHQFDHGDEICGAVVNV

**eIF4E-1** RNKGDKIALWTKNAANETAQVSIGKQWKEFLDYSNSIGFIFHDDSMRLGRGAKNRYTV

**eIF4E-2** RGKEDKIALWTKNAANETAQVSIGKQWKEFLDYSDSIDFIFHEDAERHGRGAKNRYTV

**B**

**eIF4E-2** **(T021658)** MAEESEKLRVDEVEVADDGPEEGEIVGESDDTASYLSKEIK

**eIF4E-3** **(T025160)** MAEESEKLRVDEVEVADDGPEEGEIVGESDDTASYLSKEIK

**eIF4E-^2-3^(Hybrid copy)** MAEESEKLRVDEVEVADDGPEEGEIVGESDDTASYLSKEIK

Loop I Loop II

**eIF4E-2** AKHLLENSWTFWYDK-NTKSRQAAWGSFLREVYTFSTIEDFWGVYNNINHPSKLVTGADF

**eIF4E-3** AKHPLENSWTFWFDNPTAKSRQAGWGSSLRDVYTFSTIEDFWGVYNNINHPSKLVSGADL

**eIF4E-^2-3^** AKHLLENSWTFWYDK-NTKSRQAAWGSFLREVYTFSTIEDFWGVYNNINHPSKLVTGADF

**eIF4E-2** HCFKHKIEPKWEDPVCANGGKWTMSFSKGKSDTSWLYTLLAMIGHQFDHGDEICGAVVNV

**eIF4E-3** HCFKHKIEPKWEDPVCANGGKWTMSFSKGKSDTSWLYTLLAMIGHQFDHGDEICGAVVSV

**eIF4E-^2-3^** HCFKHKIEPKWEDPVCANGGKWTMSFSKGKSDTSWLYTLLAMIGHQFDHGDEICGAVVSV

**eIF4E-2** RG---KEDKIALWTKNAANETAQVSIGKQWKEFLDYSDSIDFIFHEDAERHGRGAKNRYTV

**eIF4E-3** RAGRAKGEKIALWTKNAVNETAQVSIGKQWKEFLDYSDSIGFIFHDDAKRLDKGAKNRYTV

**eIF4E-^2-3^** RAGRAKGEKIALWTKNAVNETAQVSIGKQWKEFLDYSDSIGFIFHDDAKRLDKGAKNRYTV

**Figure S4. Amino acid sequence polymorphism between eIF4E-1, eIF4E-2, eIF4E-3 and the hybrid eIF4E-^2-3^ copy present in some ‘LD’ genotypes of *Nicotiana tabaccum*.**

The eIF4E-1 protein is encoded by the *S10760* gene (Julio et al., 2015). **A**. The amino acids that differ between eIF4E-1 and eIF4E-2 are highlighted in yellow in eIF4E-2 sequence. **B**. The amino acids that differ between eIF4E-1 and eIF4E-3 are highlighted in green. The “loop I “ and “loop II” regions contain amino acid sites known to be crucial for susceptibility to potyviruses (German-Retana *et al.*, 2008; Marcotrigiano *et al.*, 1997; Robaglia and Caranta, 2006; Bastet et al., 2018). GenBank accession numbers: *eIF4E-1* (S10760, KF155696); *eIF4E-2* (T021658, KM202068); *eIF4E-3* (T025160, KM202070); *The *eIF4E-3* sequence corresponds to the protein predicted from Edwards Reference genome (Edwards *et al.*, 2017).

**Edwards, K.D., Fernandez-Pozo, N., Drake-Stowe, K., et al.** (2017) A reference genome for Nicotiana tabacum enables map-based cloning of homeologous loci implicated in nitrogen utilization efficiency. *BMC Genomics* **18**.

**German-Retana, S., Walter, J., Doublet, B., et al.** (2008) Mutational analysis of plant cap-binding protein eIF4E reveals key amino acids involved in biochemical functions and potyvirus infection. *J Virol* **82**, 7601–12.

**Marcotrigiano, J., Gingras, A.-C., Sonenberg, N. and Burley, S.K.** (1997) Cocrystal Structure of the Messenger RNA 5’ Cap-Binding Protein (eIF4E) Bound to 7-methyl-GDP. *Cell* **89**, 951–961.

**Robaglia, C. and Caranta, C.** (2006) Translation initiation factors: a weak link in plant RNA virus infection. *Trends Plant Sci.* **11**, 40–45.
