# Supplementary material for: A complex eIF4E locus impacts the durability of va resistance to Potato virus Y in tobacco
Source: Mol Plant Pathol. 2019 May 21;20(8):1051–66. doi: 10.1111/mpp.12810 (PMC6640182; doi:10.1111/mpp.12810)
Supplement: Supplementary file 5 — Table S1 Response of va tobacco genotypes to PVY isolates. [file MPP-20-1051-s005.docx]

**Table S1. Response of *va* tobacco genotypes to PVY isolates.**

The ratios indicate the number of infected plants over the number of inoculated plants. Viral accumulation was analyzed by enzyme-linked immunosorbent assay (ELISA) in the upper non-inoculated leaves at 15 and 30 dpi. Values correspond to data obtained in two independent inoculation experiments with 6 inoculated plants per experiment. The ‘Infection rate’ corresponds to the overall percentage of infected plants for a given tobacco accession, taking into account the results obtained for all PVY isolates. Multiple *Chi-squared* tests for pairwise comparisons were performed using the *R* software v 3.2.5. Infection rates labelled with the same letter (lower cases or upper cases for infection rates observed at 15 dpi or 30 dpi respectively) are statistically identical (*P*-value < 0.05). ‘LD’ : Large Deletion ; ‘SD‘ : Small Deletion ; ‘Fs’ : Frameshift ; ‘EMS’ : ‘EMS mutants’ ; Sus’: susceptible genotype.
