# Supplementary material for: A complex eIF4E locus impacts the durability of va resistance to Potato virus Y in tobacco
Source: Mol Plant Pathol. 2019 May 21;20(8):1051–66. doi: 10.1111/mpp.12810 (PMC6640182; doi:10.1111/mpp.12810)
Supplement: Supplementary file 6 — Table S2 Response of representative va tobacco genotypes to various PVY isolates. [file MPP-20-1051-s006.docx]

**Table S2. Response of representative *va* tobacco genotypes to various PVY isolates.**

The ratios indicate the number of infected plants over the number of inoculated plants. Viral accumulation was analyzed by enzyme-linked immunosorbent assay (ELISA) in the upper non-inoculated leaves at 18 dpi or 30 dpi (*). The ‘Overall infection rate’ indicates the percentage of infected plants for a given tobacco accession, taking into account all PVY isolates used. Multiple *Chi-squared* tests for pairwise comparisons were performed using the R software v 3.2.5. Tobacco genotypes infection rates labelled with the same letter are statistically identical (*P*-value < 0.05). ‘LD’: Large Deletion; ‘SD‘ : Small Deletion ; ‘Fs’: ‘Frameshift’; ‘EMS’ : ‘EMS mutants’; Sus’: susceptible genotype. SON41, Alger1, Marti3, LYE84.2, CAA157, CAA141, CAA16 and LYE72-Puc2Pl3 isolates belong to the clade C group C1, and Cadgen and LYE90v to group C2. CAA156 and LYE245 isolates are PVY recombinants between O and C clades. Other isolates belong to the group PVYNTN (NTN-H) and PVYWi (WilgaP) (Glais *et al.*, 2002). PVY isolates belonging to the N clade are ‘Pologne 6 puc3 pl2’ and ‘N605’, and one isolate comes from Brazil, ‘Bresil 1054’. Some of the isolates are referenced in Ben Khalifa *et al.* (2012); Moury *et al.* (2004); Moury, (2010); Moury *et al.* (2011); Woloshuk *et al.* (1993).
