# Supplementary material for: A complex eIF4E locus impacts the durability of va resistance to Potato virus Y in tobacco
Source: Mol Plant Pathol. 2019 May 21;20(8):1051–66. doi: 10.1111/mpp.12810 (PMC6640182; doi:10.1111/mpp.12810)
Supplement: Supplementary file 7 — Table S3 Amino acid changes in the VPg central region (amino acids 101 123) of the progenies of five PVYN isolates, following infection of 13 different va tobacco accessions, in comparison with the sequence of the parental isolates. [file MPP-20-1051-s007.docx]

**Table S3. Amino acid changes in the VPg central region (aa 101-123) of the progenies of five PVY^N^ isolates, following infection of 13 different *va* tobacco accessions, in comparison with the sequence of the parental isolates.**

The amino acid positions are numbered according to the VPg sequence of PVY-N605 (GenBank X97895). All five PVY^N^ isolates (LA7, 11.08, CSA1, CSA6 and MaSan4) display amino acids 101S, 105K, 108V, 109E and 119G before their propagation in the *va* tobaccos. The exponent numbers (^1, 2^ or ^3^) close to PVY isolates names refer to the numbers of independent progenies sequenced. ‘Total sequences (RB)’ does not include the 20 progenies sequences obtained in BB16. C = cysteine, D = aspartic acid, E = glutamic acid, G = glycine, I = isoleucine, K = lysine, M = methionine, N = asparagine, Q = glutamine, S = serine, T = threonine, and V = valine. ’–‘ indicates no mutation. “All isolates” indicates that for all PVY^N^ isolates no mutation in the VPg appeared following propagation in the BB16 susceptible tobacco.
