# Supplementary material for: A complex eIF4E locus impacts the durability of va resistance to Potato virus Y in tobacco
Source: Mol Plant Pathol. 2019 May 21;20(8):1051–66. doi: 10.1111/mpp.12810 (PMC6640182; doi:10.1111/mpp.12810)
Supplement: Supplementary file 8 — Table S4 Amino acid changes in the VPg central region (amino acids 101 123) of the progenies of four PVYO isolates, following infection of 13 different va tobacco accessions, in comparison with sequence of the parental isolates. [file MPP-20-1051-s008.docx]

**Table S4. Amino acid changes in the VPg central region (aa 101-123) of the progenies of four PVY^O^ isolates, following infection of 13 different *va* tobacco accessions, in comparison with sequence of the parental isolates.**

The amino acid positions are numbered according to the VPg sequence of PVY-O (GenBank U09509). All the four PVY^O^ isolates (O139, SAV8, SN3 and LA4) display amino acids 101S, 105K, 119G, 120S and 121N, before their propagation in the *va* tobaccos. The exponent numbers (^1, 2^ or ^3^) close to PVY isolates names refer to the numbers of independent progenies sequenced. *Total sequences (RB) does not include the 20 progenies sequences obtained in BB16. E = glutamic acid, G = glycine, H = histidine, K = lysine, M = methionine, N = asparagine, Q = glutamine, R = arginine, S = serine, T = threonine, V = valine, Y=tyrosine.’–‘ indicates no mutation detected in the progenies. “All isolates” indicates that for all PVY^O^ isolates no mutation appeared in the VPg following propagation in the BB16 susceptible tobacco.
