# Supplementary material for: A complex eIF4E locus impacts the durability of va resistance to Potato virus Y in tobacco
Source: Mol Plant Pathol. 2019 May 21;20(8):1051–66. doi: 10.1111/mpp.12810 (PMC6640182; doi:10.1111/mpp.12810)
Supplement: Supplementary file 9 — Table S5 List of primers and probes used in this study. [file MPP-20-1051-s009.docx]

**Table S5: List of primers and probes used in this study.**

| **Primer name** | | **Sequence (5’-3’)** |
| --- | --- | --- |
| **Quantification of viral load** | | |
| FAM-TAMRA Probe | TGATGAATGGGCTTATGGTTTGGTGCA | |
| 5'-UnivF | CATAGGAGAAACTGAGATGCCAACT | |
| 5'-UnivR | TGGCGAGGTTCCATTTTCA | |
| **Analysis of the sequence of the VPg protein of the viral progeny** | | |
| 3'NTR | GTCTCCTGATTGAAGTTTAC | |
| RJ2-F | GATCTCAAGTTGAAGGGGAC | |
| NIa-R | AYGCAGARTGYTTAGCTTGC | |
| **Analysis of *eIF4E-2* gene expression by Quantitative RT-PCR** | | |
| eIF4E-2 Fwd (=T021658E6TF) | gcaagttagttacgggagca | |
| eIF4E-2 Rev | CCTTACCTCGGACATTAACT | |
| RL2-Fw | GGCGAAATGGGTCGTTTGATC | |
| RL2-Rev | CGTTCCGTTCGCCGAAGTCG | |
| ef1-alpha-Fw | ATTGGAAATGGATATGCTCCA | |
| ef1-alpha-Rev | TCCTTACCAGAACGCCTGTC | |
| **Mutants screening for *eIF4E-2* and *eIF4E-3* genes** | | |
| T025160F6 | GTTAGTTTCCGGAGCAGACTTGCA | |
| T025160R6-2 | CGGCCAGCACGGACACTAA | |
| T021658E6TF | GCAAGTTAGTTACGGGAGCA | |
| T021658E3T2R | CTCCTTACCTCGGACATTAACT | |
| **Copy number analyses** | | |
| E2-E2E3F | AAATCGTGGGGGAATCAGATG | |
| E2-E2E3R | AACTTCGCGAAGGAAGCTAC | |
| Probe E2-E2E3 | FAM-ATACGGCGTCGTATTTGAGC-MGB | |
| E2F | GCAAGTTAGTTACGGGAGCA | |
| E2R | AATATCCTCAGCATACCGTG | |
| Probe E2 | FAM-AGCCAAAGTGGGAAGATCCT-MGB | |
| E3-E2E3F | TTCCCCTTGGTATTTTCTTAAC | |
| E3-E2E3R | CGGCCAGCACGGACACTAA | |
| Probe E3-E2E3 | FAM-CTGGCAATGATTGGACATCA-MGB | |
| NR4-F | GCCATCTCACACAACACTGG | |
| NR4-R | TGTCATAGCCCATCTTCTCCA | |
| Probe NR4 | VIC-CTTTGGCTTGTGGACCACCTCCTATGA-MGB | |
