# Supplementary material for: A complex eIF4E locus impacts the durability of va resistance to Potato virus Y in tobacco
Source: Mol Plant Pathol. 2019 May 21;20(8):1051–66. doi: 10.1111/mpp.12810 (PMC6640182; doi:10.1111/mpp.12810)
Supplement: Supplementary file 10 — Table S6 The deletion at the locus eIF4E‐1LD on chromosome 21 co‐segregates with the resistance durability character. [file MPP-20-1051-s010.docx]

**Table S6. The deletion at the locus *eIF4E-1^LD^* on chromosome 21 co-segregates with the resistance durability character**

|  |  | **Genotype** | | |  | **Phenotype** |  | ***P*-value (*Chi^2^*)** |
| --- | --- | --- | --- | --- | --- | --- | --- | --- |
|  |  | ***eIF4E-3 locus*** |  | ***eIF4E-1 locus*** |  |  |  |  |
| **VAM** |  | *eIF4E-^2-3^/ eIF4E-^2-3^* |  | *eIF4E-1^LD^*/*eIF4E-1^LD^* |  | 6% (2/33) |  |  |
| **EMS1** |  | *eIF4E-3/eIF4E-3* |  | *eIF4E-1^KO^*/*eIF4E-1^KO^* |  | 98% (32/33) |  |  |
| **F1** |  | *eIF4E-3/ eIF4E-^2-3^* |  | *eIF4E-1^LD^*/*eIF4E-1^KO^* |  | 12% (4/33) |  | 0.67 ^$^ |
| **F2** |  | *eIF4E-^2-3^/ eIF4E-^2-3^* |  | *eIF4E-1^LD^*/*eIF4E-1^LD^* |  | 10% (7/68) |  | 0,78 ^†^ |
|  |  | *eIF4E-3/eIF4E-3* |  |  |  |  |  |  |
|  |  | *eIF4E-3/ eIF4E-^2-3^* |  |  |  |  |  |  |
|  |  | *eIF4E-^2-3^/ eIF4E-^2-3^* |  | *eIF4E-1^KO^*/*eIF4E-1^KO^* |  | 47% (31/66) |  | 4.6x10^-6^ ^‡^ |
|  |  | *eIF4E-3/eIF4E-3* |  |  |  |  |  |  |
|  |  | *eIF4E-3/ eIF4E-^2-3^* |  |  |  |  |  |  |
|  |  | *eIF4E-^2-3^/ eIF4E-^2-3^* |  | *eIF4E-1^LD^*/*eIF4E-1^KO^* |  |  |  |  |
|  |  | *eIF4E-3/eIF4E-3* |  |  |  | 18% (22/122) |  | 0,63 ^§^ |
|  |  | *eIF4E-3/ eIF4E-^2-3^* |  |  |  |  |  |  |

F2 population (256 plants) from the cross (VAM x EMS1) segregating for the deletion *eIF4E-1^LD^* were inoculated with PVY-O139. The allele *eIF4E-^2-3^* corresponds to the hybrid form *eIF4E-^2-3^* on chromosome 14 as present in the VAM accession. The phenotype corresponds to the infection ratio (number of infected plants / number of inoculated plants) estimated by ELISA at 30 dpi. χ2 statistic tests were used to investigate whether the resistance durability phenotype (estimated as the infection rates) differed between ($) F1 plants and the parental VAM; (†) F2 plants homozygous *eIF4E-1^LD^*/*eIF4E-1^LD^* and the parental VAM; (‡) F2 plants homozygous *eIF4E-1^KO^*/*eIF4E-1^KO^* and the parental EMS1; (§) F2 plants heterozygous *eIF4E-1^LD^*/*eIF4E-1 ^KO^* and the F1 progeny. The χ2 test was used to test the null hypothesis, stating that there is no significant difference between the expected and observed result. By statistical convention, we used the 0.05 probability level as our critical value.
